# Carotid Velocities Determine Cerebral Blood Flow Deficits in Elderly Men with Carotid Stenosis <50%

**DOI:** 10.1155/2012/579531

**Published:** 2012-06-19

**Authors:** Arkadiusz Siennicki-Lantz, Per Wollmer, Sölve Elmståhl

**Affiliations:** ^1^Division of Geriatric Medicine, Department of Health Sciences, Skane University Hospital in Malmö, Lund University, 205 02 Malmö, Sweden; ^2^Unit of Clinical Physiology and Nuclear Medicine, Department of Clinical Sciences, Skane University Hospital in Malmö, Lund University, 205 02 Malmö, Sweden

## Abstract

To examine if mild carotid stenosis correlates with silent vascular brain changes, we studied a prospective population-based cohort “Men born in 1914.” Data from followups at ages 68 and 81, have been used. Carotid ultrasound was performed at age 81, and cerebral blood flow (CBF) was measured with SPECT at age 82. Out of 123 stroke-free patients, carotid stenosis <50% was observed in 94% in the right and 89% in the left internal carotid arteries (ICAs). In these subjects, Peak Systolic Velocities in ICA correlated negatively with CBF in a majority of several brain areas, especially in mesial temporal area. Results were limited to normotensive until their seventies, who developed late-onset hypertension with a subsequent blood pressure, pulse pressure, and ankle-brachial index growth. Elderly with asymptomatic carotid stenosis <50% and peak systolic velocities in ICA 0.7–1.3 m/s, should be offered an intensified pharmacotherapy to prevent stroke or silent cerebrovascular events.

## 1. Introduction

Stroke risk in patients with symptomatic internal carotid artery (ICA) stenosis greater than 70% can be reduced effectively by carotid endarterectomy. In most cases, carotid ultrasound is performed in association with TIA or stroke and, in case of significant stenosis, an operative treatment or pharmacotherapy is initiated as a secondary prevention. Additionally, asymptomatic patients with carotid stenosis could benefit from primary preventive operative treatment with carotid endarterectomy in presence of a hemodynamically significant 60–99% ICA stenosis [1–3]. A number of patients needed to be treated with this method are approximately 20, to prevent one stroke in 5 years. Even in mild carotid stenosis (<50%), the rate of stroke increased to 13% at 7.7 years in elderly patients [4]. Silent embolic infarcts predict also future ipsilateral stroke in at least moderate asymptomatic carotid stenosis [5], and elderly with moderate carotid stenosis had a significant ipsilateral cerebral perfusion delay on MRI [6]. It is still unclear to what extent the mild carotid stenosis is responsible for, or correlates with silent vascular brain changes.

Calculating carotid stenosis to less than 50% could be made by radiologic or sonographic methods. Measurement of peak systolic velocities (PSVs) in ICA has been used worldwide, and maximal PSV, defined between 1.25–1.4 m/s, was used delimitate stenosis to maximum 50%, sometimes with mandatory plaque or intimal thickening visible [7]. When estimating cerebral blood supply and carotid stenosis with ultrasound in the elderly, one must be aware, that age and DBP-level decrease PSV, while SBP and pulse pressure increase PSV in elderly [8, 9]. In elderly with cerebral ischemia, larger diameters of common carotid artery and lower PSV have been observed, probably due to increased intracerebral circulatory resistance [10, 11]. At the same time, the risk of stroke was highest in subjects with increased PSV in middle cerebral artery [12]. Changes in blood pressure in the very elderly could also affect PSV, mainly due to a frequently observed blood pressure decline after age 75, or decreased cardiac dynamic parameters.

The aim of our study was to estimate the presence of silent changes in cerebral blood flow (CBF) in a populations sample of stroke-free elderly men without carotid stenosis or less than 50% and to examine if there was a dose-response relationship between PSV in ICA and a grade of CBF decline. 

## 2. Methods

### 2.1. Study Sample

**Figure 1:**
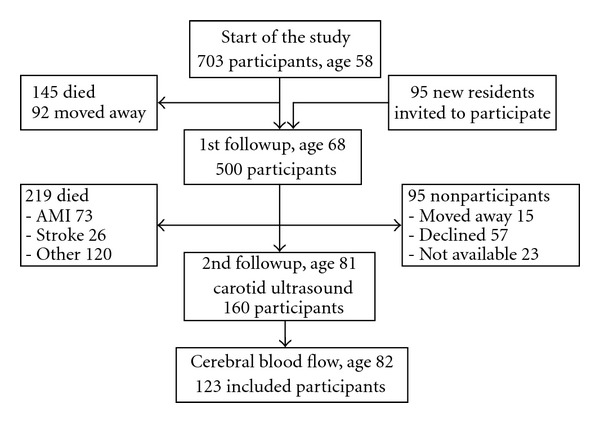
Followup of the cohort “Men born in 1914.”

 A prospective population sample study, “Men born in 1914,” has been in progress since 1968. It includes all men born in the even months of 1914 in the city of Malmö, Sweden. A total of 809 men were invited to participate in the study, and 703 men took part in the first health examination. When they were 68 years old, 465 men in the cohort and an additional 95 new residents were invited to attend a new examination. Five hundred of them agreed to participate in this first followup (Figure 1). The most recent/second followup of the cohort started when the subjects reached 81 years of age, and 281 men were found to be still alive. Of these, 185 agreed to take part (66%) in a new investigation, including both physical and psychological examinations as well as carotid ultrasound bilaterally. One year later, all 185 subjects were invited to a measurement of cerebral blood flow with SPECT, but only 129 of them agreed to participate. Six subjects had suffered from a stroke previously, according to the local stroke register, and were excluded from the study. Finally, 123 participants were included into the analysis.

### 2.2. Health Examination

Vascular risk factors, including hypertension, levels of blood glucose, cholesterol, and triglycerides during fasting conditions as well as body mass index (BMI) were measured at age 68. Blood pressure was measured sphygmomanometrically with Korotkoff method in the upper right arm in the supine position after 15 min of rest (at age 68) and in the sitting position (at age 81), using a calibrated mercury manometer and rubber cuffs (12 × 35 cm for normal and 15 cm for obese subjects). The participants were also classified as nonsmokers/former smokers and current smokers. Peripheral circulation in the lower extremities was estimated using the ankle-brachial pressure index (ABI) at age 68 and 81 (see below). At the recent followup at age 81, the medical examination was repeated, and 185 men answered a questionnaire focusing on lifestyle and health markers. Possible dementia was classified according to the DSM-IV criteria and, according to them, one subject was diagnosed as being demented. The Mini-Mental State Examination (MMSE) was performed on 171 men, giving a mean value of 28.23 ± 1.85  (SD); range 18–30. Eight subjects had MMSE scores ≤24. Hypertension was defined as systolic and diastolic brachial BP ≥ 160 mmHg or ≥ 90 mmHg, respectively, or medication for hypertension. These hypertension criteria have been used previously and were valid until the World Health Organization drew up new ones in 1999. All the subjects had been monitored and treated during their lifetime according to these hypertension criteria, and therefore they were used for the statistical analysis.

### 2.3. Carotid Duplex Ultrasonography

The carotid arteries were examined at age 81, using a computed sonography system (Acuson XP 10, Acuson, Mountain View, CA, USA) with a 7 MHz B-mode real-time linear scanner, including a 5 MHz pulsed and color-coded Doppler. The color-coded Doppler was used to identify areas with high flow velocities in the internal carotid artery (ICA), and the maximum flow velocity (m/s) was measured with the pulsed Doppler. The angle between flow direction and Doppler signal was carefully corrected and always kept below 65°. The degree of stenosis in the ICA was determined from the peak systolic velocity to the equation:  *y* = 0.54 · *e* ^0.021*x*^, where  *y*  is the peak systolic velocity in the ICA in m/s, and  *x*  is the degree of stenosis expressed as the diameter reduction in percentage. Diameter reduction in percent = [*b* − *a*] · 100, where “*a*” is the smallest diameter in the stenotic zone, and “*b*” is the diameter of normal common carotid artery proximal to the stenosis [13]. 

### 2.4. Cerebral Blood Flow Estimation

**Figure 2:**
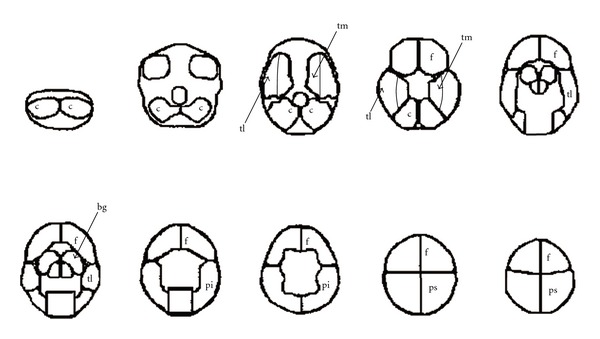
Distribution of regions of interest used for cerebral blood flow estimation. (f) Frontal; (tl) temporal lateral and (tm) temporal mesial; (pi) parietal inferior and (ps) parietal superior ROI; (bg) basal ganglia; (c) reference region in cerebellum.

Each subject received an intravenous injection of 800 MBq 99mTc-HMPAO (Ceretec; Amersham Inc., Little Chalfont, Buckinghamshire, UK). The acquisition was performed under resting conditions on a triple-headed gamma camera system (Siemens Multispect 3, Siemens, Chicago, IL, USA) with fan beam low-energy collimators and in 360° rotation (64 views, 20 s/view, in a  128 × 128  matrix, and a zoom factor of 1.23). The energy window used had a 15% window centered over the 140 keV peak. Image processing included reconstruction of 10 transaxial 1 cm-thick slices, from 1 cm below the orbitomeatal line and upwards. Regions of interest were delineated in each slice and defined as right or left: frontal, temporal medial, temporal lateral, parietal superior, parietal inferior, basal ganglia, and subcortical as in Figure 2. CBF was expressed as a regional count density percentage of the mean cerebellar count density.

### 2.5. Peripheral Arterial Circulation

Ankle blood pressure was estimated, at the ages of 68 and 81 years, by placing a cuff around the ankle and using a Doppler signal on the tibial posterior artery or dorsal foot artery to detect peripheral blood flow. Both ankle pressure and the reference pressure in the arm were calculated using the strain gauge system. The occluding cuff was placed on the arm, and finger blood flow was continuously recorded by mercury-in-silastic strain gauge pulse sensor placed on the proximal phalanx of the first digit. Wheatstone bridge with amplifier was used to record the resistance of the strain gauges, and a pressure transducer (Siemens-Elema EMT 746 with amplifier EMT 311) was used to record cuff pressures. Arithmetic average of a two recordings was used. The ankle-brachial pressure index (ABI) was calculated for each leg by dividing the ankle SBP by the highest individual upper arm SBP value. 

### 2.6. Statistical Analysis

Summary values were expressed as means ± standard deviation. Correlation analyses were performed using the Spearman correlation tests. Partial correlation has been calculated between variables when controlling for the grade of carotis stenosis. The Mann-Whitney  *U*  test for independent samples was used to analyze differences. A two-tailed *P* value of less than 0.05 was considered statistically significant. All data analyses and statistical calculations were performed using the SPSS (SPSS Inc., Chicago, USA) data package. The study was approved by the local ethics committee at Lund University (LU 111-82). All subjects gave their informed consent. 

## 3. Results

The data of the cohort “Men born in 1914” are presented in Table 1. Carotid stenosis ≥50% was more frequent in the right ICA than on the left side (11.6% versus 5.7%), and mean PSV was also higher in the left than in right ICA. However, the vast majority of this population sample was composed of subjects with carotid stenosis less than 50%, and this subgroup has been chosen for further analyses. In subjects with carotid stenosis <50% on the right side, 22 were nonstenotic out of the 116, while on the left side, 21 were nonstenotic out of the 110 subjects. Blood pressure data, between the first followup at age 68 and at the end of the study at age 81, showed an overall SBP decline with mean 7.2 mmHg, and DBP decline with 9.6 mmHg. Pulse pressure slightly increased with 2 mmHg. Ankle-brachial index (ABI) decreased with mean index  .10 in the right and  .09 in the left leg during the followup.

**Figure 3:**
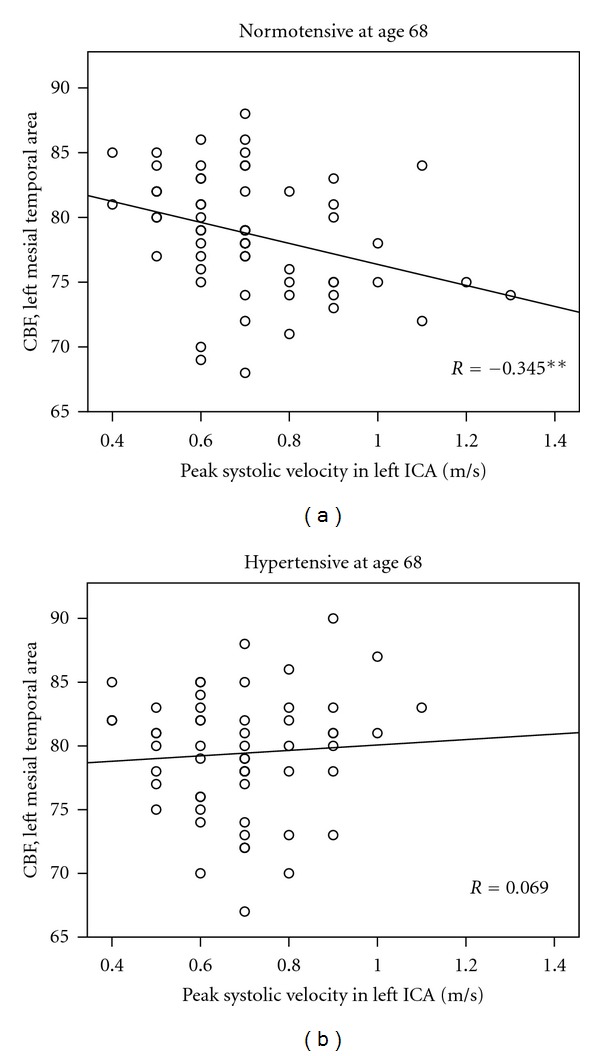
Distribution of cerebral blood flow values (CBF), estimated in left mesial temporal area, depending on the level of peak systolic Velocity in left internal carotid artery (ICA). scatter presented for both normotensive and hypertensive subjects at age 68.

In a whole cohort, CBF values did not correlate with carotid PSV. The presence of long-term/early hypertension has been chosen as a grouping factor, and the cohort has been divided into hypertensive and normotensive subgroup at age 68. In subjects who were found normotensive at 68, both left and right carotid PSV at age 81 correlated negatively with CBF in several brain areas. In 110 subjects with left-sided carotid stenosis <50%, 59 were normotensives, and negative correlation has been observed between PSV in left ICA and CBF in frontal, mesial temporal, inferior and superior parietal and subcortical areas (Table 2, 1st column). In the 57 normotensives with right-sided carotid stenosis <50%, PSV in left ICA correlated negatively with CBF in frontal, mesial temporal, inferior and superior parietal, and subcortical areas as well as in basal ganglia (Table 2, 2nd column). In both carotids, highest correlation coefficients were observed with CBF in mesial temporal areas, bilaterally (Figure 3). In those who were hypertensive at age 68, no significant correlations have been observed between PSVs and regional CBF, either on the left side (56 subjects with left ICA stenosis <50%) or the right side (59 subjects with right ICA stenosis <50%) (Table 2, columns 3-4). To analyse if the relationship between PSV and CBF is independent in carotid stenosis, a partial correlation has been calculated between PSV and CBF controlling for carotis stenosis in %. The data showed a nonaltered results for right ICA and moderately diminished scope for left ICA (significant for 3 instead of 8 brain areas) (Table 3). Similarly, as in nonadjusted analysis, no significant correlations have been observed between PSV and CBF in hypertensives at age 68 after controlling for carotid stenosis.

To explain the different trends in PSV-CBF relationship by the presence of an early hypertension, vascular characteristics in the normotensive and hypertensive subgroups have been analysed. PSVs did not differ significantly between normotensive and hypertensive subjects (left ICA: 0.71 ± 0.18 versus 0.69 ± 0.16, *P* = 0.455; right ICA: 0.67 ± 0.19 versus  0.67 ± 0.16,  *P* = 0.978). When comparing PSVs in all 160 survivors, who were examined by ultrasound but before inclusion in the CBF, normotensive subjects presented significantly higher PSVs in left ICA (0.72 ± 0.18 versus 0.67 ± 0.16, *P* = 0.035) and slightly larger, but nonsignificant, difference in right ICA (0.69 ± 0.19 versus 0.65 ± 0.16, *P* = 0.213). In normotensive subjects at age 68, left-sided PSV correlated positively with SBP at age 81 and with time difference in SBP between ages 81 and 68, showing highest left-sided PSV values in those subjects who developed largest SBP-increase during 13-year followup (Table 4). This relationship was not observed in hypertensive subgroup at age 68. Pulse pressure levels at age 81 correlated strongly positively with left-sided PSVs on both sides in normotensive, but not in hypertensive subgroup. PSV correlated also negatively with ABI in both legs at age 81, both in normotensive (left ICA) and hypertensive at age 68 (right ICA). Also a lower ABI, estimated at age 68, predicted higher PSV values in both subgroups. The time trend of ABI, expressed as a difference between individual values measured at 81 and 68, correlated negatively with PSV, that is the larger decline in ABI over observation time, the higher were the PSVs (Table 4).

 Subjects who were normotensive already at age 68, differed from hypertensive ones, by lower pulse pressure at 68 (52.1 versus 64.4 mmHg;  *P* < 0.001), and at 81 (58.1 versus 63.8 mmHg; *P* = 0.015). Normotensives had also more stable blood pressure during 13-year followup, compared to a marked blood pressure decline in hypertensives (SBP difference age 81–68. Normotensives: 1.5 ± 17.5 mmHg versus hyper-tensives: −14.9 ± 21.2 mmHg;  *P* < 0.001) (DBP difference age 81–68. Normotensives: −4.4 ± 8.6 versus hypertensives: −14.4 ± 8.5 mmHg*; P* < 0.001). ABI at age 68 did not differ between normo- and hyper-tensives, but at age 81, ABI was higher in normotensives in the left leg (1.04 ± 0.22 versus  0.95 ± .17;  *P* = 0.005), and ABI time decline from age 68 to 81 was lower than in hypertensives (left ABI difference, age 81–68:  −0.05 ± 0.13 versus −0.13 ± 0.16;  *P* = 0.007). Values of CBF did not differ between normotensive and hypertensive subjects diagnosed at age 68 (Table 5).

## 4. Discussion

In our study cohort, comprising 123 stroke-free men who reached the age of 82 years and performed CBF measurement, the frequency of a moderate- or high-grade carotid stenosis was very low. Survival analysis performed previously in this cohort showed an association between early-onset carotid stenosis at age 68 with a higher mortality from ischaemic heart disease during following ten years, especially when combined with low ABI [14]. Then, due to the selective mortality, the population-based sample of octogenarians studied here has low frequency of high-grade carotid stenosis. Some of the disabled elderly chose not to join the last reexamination or were not capable to be examined due to disabilities or hospitalization. A dropout analysis has been published earlier, and the background data did not differ between these groups concerning: SBP, DBP, presence of hypertension, ankle-arm indexes, B-glucose, triglycerides and cholesterol levels, prevalence of accumulated vascular events, smoking history at age of 68 years; prevalence of stroke up to age of 82 years, or difference in SBP or DBP between ages 68 and 82 years. Significant moderate difference has only been observed in BMI at age 68 [15]. A limitation of this study is a restriction to male subjects. However, study subjects were invited in a randomized way and are fairly representative of the men of their age in the community. Our findings ignore also a possible confounding effect of antihypertensive medication, when comparing hyper- and normo-tensives.

It is unclear if increased PSV in ICA should be regarded as a risk factor for silent brain damage, or if it is a reverse marker of increased cerebrovascular resistance. Generally, low, not high ICA flow velocities are treated as indicators of higher cerebral vascular resistance [16]. In acute as well as chronic stable patients with ischemic stroke, there were significantly lower PSVs and higher resistance index compared to the nonstroke patients, independently of extracranial carotid atherosclerosis [9, 10]. On the other side, in subjects with PSV < 1.25 m/s and the presence of a sonographic atherosclerotic plaque <50%, a significantly higher frequency of both 1-, 2- and 3-vessel coronary artery disease has been observed [17]. 

In the common carotid, internal carotid and the vertebral arteries flow velocities decrease during ageing. The luminal diameter has been reported as constant or increasing with age. This age-related enlargement in cross-sectional area results from stretching of elastic fibers in the vessel wall or an altered baroreceptor reflex. Parallely, an age-related decline of intravascular flow volume was observed in the ICA. Due to a pronounced decrease in end-diastolic flow velocity, the resistance index decreased during ageing [18]. While ICA's PSVs are generally decreasing with age, the role of hypertension is contradictory. In a majority of studies, an enhancing effect of hypertension was observed [8]. Except inverse correlation with age, peak common carotid artery velocity in the elderly people was also inversely associated with diastolic blood pressure and directly associated with pulse pressure [9], while ICA PSVs decrease with advancing age and increase with increasing pulse pressure [8]. As the effects of systolic and diastolic pressure seems to be opposing, pulse pressure, which summarize their combined effect, is related to PSV increase by 0.21 cm/sec/mm [8]. In our study, high PSVs correlate well with pulse pressure. PSV correlated also with ABI, and was higher in subjects who had mostly aggravating atherosclerotic process in peripheral arteries, expressed by decreasing ABI over the last 13 years. During early examination of the same cohort, an ABI < 0.9 was associated with a 2.4 times higher total mortality and a 2.0 times higher cardiac event rate [19]. Knowing that high pulse-pressure is a risk factor for end-organ damage, that is cerebrovascular disease, in our cohort, high PSV in subjects with low CBF can reflect deleterious effect of high pulse pressure and/or express vascular stiffness. Knowing that PSVs generally decrease with age, in some very elderly, an effect of arterial stiffness expressed by high pulse pressure may reverse this trend and lead to higher PSVs, or just erase an age-related PSV decline by reflecting higher atherosclerotic load in intracranial vessels and in CBF. As speculated [8], the autoregulatory mechanisms of CBF could blunt velocity changes in the carotid artery to maintain a constant pressure gradient between the cerebral arteries and the brain. In case of high pulse pressure, cerebral vasoconstriction may be down-regulated to maintain flow during the longer lower-pressure diastolic phase of the cardiac cycle. The net effect of this decrease in downstream resistance would be to enhance flow and increase velocities in the setting of an elevated pulse pressure. The effect of pulse pressure must overcome higher cerebral vascular resistance, which is expressed by low, not high, ICA flow velocities [16]. It has also been reported that, in acute as well as chronic stable patients with ischemic stroke, there were significantly lower flow velocities and higher resistance index compared to the nonstroke patients, independently of extracranial carotid atherosclerosis [9, 10]. 

Cardiac function affect PSV by a direct correlation between PSV in the left CCA and ICA and fraction shortening of heart muscle, and between PSV in the left CCA and peak velocity on left ventricular outflow tract [20]. Assuming that heart failure is more frequent in subjects with cerebrovascular disease, the PSVs should have an opposite relation to CBF than observed in this study. Probably, in relatively healthy octogenarians of this cohort, this mechanism was attenuated by presence of heart failure in subjects who were still normotensive at age 68 and who developed a mild hypertension at age 81, while absent in those who had an early hypertension, reached an inflection point in their seventies and expressed lower blood pressure levels at the time of ultrasound examination [21]. In our cohort, PSV values were higher on the left side in normotensives at age 68, who in their majority developed blood pressure increase during followup, compared to hypertensives whose blood pressure generally declined until age 81. Highest PSV values have also been observed in those subjects who had high SBP at age 81, and most increasing SBP values during last 13 years. These findings were limited to subjects who were normotensive at the beginning of the observation at age 68. This subgroup had also equal values of decreasing and increasing SBP during followup, while hypertensive at 68 registered predominant blood pressure decline until age 81, especially concerning DBP. In hypertensives at age 68, contrary to normotensives, PSVs were even negatively correlated with DBP values, as it was observed in other studies [8]. Decreasing blood pressure and lower PSVs in formerly hypertensives could reflect incipient heart failure or dysautonomia.

 On the whole, these results suggest that our population of octogenarian men consists of two different groups concerning blood pressure trend, resulting in a different cause-effect relationship between ICA's PSVs and regional CBF. Firstly, elderly with late-onset hypertension (after 68 years) with increasing SBP values over time had slightly higher PSV, mainly on the left side and a clear relationship between high PSV in carotids, the grade of systolic blood pressure increase and CBF defects of arteriosclerotic origin probably due to dynamic effect of high pulse pressure, which simultaneously affects cerebral circulation. Secondly, subjects with early-onset hypertension, with blood pressure decline over time, either due to dysautonomia or heart failure, where lower blood pressure in the eighth decade express higher vascular lifetime-load, and where higher peripheral resistance in cerebral vessels and lower cardiac output could blur the pulse pressure related PSV increase and its effect on CBF [22]. 

## 5. Conclusion

To our knowledge, this is a first study showing a correlation between high peak systolic velocities in the internal carotid artery and a widespread cerebral blood flow defects in stroke-free very elderly men with carotid stenosis less than 50%. This observation was limited to those who were normotensive and untreated until their seventies and in majority developed late-onset hypertension with subsequent blood pressure and pulse pressure growth. Elderly men with velocities between 0.7–1.3 m/s should be offered an intensified pharmacotherapy and lifestyle modification. 

## Figures and Tables

**Table 1:** The background data of the 123 subjects collected at the 1st and the 2nd followup of the cohort.

	Age 68 years	Age 81 years
In whole cohort:	*n* = 123	*n* = 123
carotid stenosis right <50%, *n* (%)		116 (94.3)
carotid stenosis left <50%, *n* (%)		110 (89.4)
right ICA's PSV (m/s); min–max; mean (SD)		.3–3.0; .76 (4.1)
left ICA's PSV (m/s); min–max; mean (SD)		.4–4.0; .82 (.47)

In subjects with right carotid stenosis <50%:		*n* = 116
right ICA's PSV (m/s); min–max; mean (SD)		.3–1.2; .67 (.17)
frequency of stenosis: 0%–35%–40%; *n* subjects		22–91–3

In subjects with left carotid stenosis <50%:		*n* = 110
Left ICA's PSV (m/s); min-max; mean (SD)		.4–1.3; .69 (.17)
Frequency of stenosis: 0%–35%–40%–45%; *n* subjects		21–85–2–2

In subjects with carotid stenosis <50%, left or right:	*n* = 107	*n* = 107
SBP, mmHg (SD)	150.6 (19.0)	143.4 (15.3)
DBP, mmHg (SD)	92.1 (7.9)	82.5 (6.4)
pulse pressure, mmHg (SD)	58.1 (14.8)	60.1 (14.1)
Ankle brachial index:		
Right	1.13 (.12)	1.03 (.19)
Left	1.09 (.12)	1.00 (.19)
Difference ABI ages 81–68, right		−.10 (.17)
Difference ABI ages 81–68, left		−.09 (.15)

ICA: internal carotid artery.

**Table 2:** Correlation coefficients between peak systolic velocity in right/left ICA and cerebral blood flow estimated in several regions of interest. Analyses performed in study subjects defined as normotensive and hypertensive at age 68.

	Normotensive at age 68	Hypertensive at age 68	Normotensive at age 68	Hypertensive at age 68
	ICA left	ICA right	ICA left	ICA right
	*N* = 55	*N* = 57	*N* = 55	*N* = 59
Frontal				
R	−.244	−.333^∗^	−.014	.106
L	−.273^∗^	−.282^∗^	.064	.079
Mesial temporal				
R	−.345^∗∗^	−.286^∗^	.069	.140
L	−.369^∗∗^	−.376^∗∗^	.030	.165
Lateral temporal				
R	−.237	−.186	−.013	.218
L	−.214	−.252	.040	.171
Superior parietal				
R	−.355^∗∗^	−.279^∗^	.001	.088
L	−.312^∗^	−.217	.140	.181
Inferior parietal				
R	−.294^∗^	−.355^∗∗^	.026	.083
L	−.282^∗^	−.270^∗^	.146	.123
Basal ganglia				
R	−.138	−.261^∗^	.007	.043
L	−.191	−.234	.065	.036
Subcortical area	−.309^∗^	−.337^∗^	.045	.066

^∗^Correlation is significant at  .05 level (2-tailed). ^∗∗^Correlation is significant at  .01 level (2-tailed). ICA: internal carotid artery.

**Table 3:** Partial correlation coefficients between peak systolic velocity in right/left ICA and cerebral blood flow, controlled for the grade of carotid stenosis ipsilaterally.

	Normotensive at age 68	
	ICA left	ICA right
	*N* = 55	*N* = 57
Frontal		
R	−.149	−.347^∗∗^
L	−.156	−.333^∗^
Mesial temporal		
R	−.284^∗^	−.286^∗^
L	−.278^∗^	−.271^∗^
Lateral temporal		
R	−.203	−.221
L	−.159	−.248
Superior parietal		
R	−.270^∗^	−.329^∗^
L	−.219	−.276^∗^
Inferior parietal		
R	−.213	−.341^∗^
L	−.173	−.300^∗^
Basal ganglia		
R	−.137	−.240
L	−.194	−.216
Subcortical area	−.226	−.337^∗^

**Table 4:** Correlation coefficients between peak systolic velocity in right/left ICA and blood pressure trend. Ankle-brachial index (ABI) at ages 68, 81, and its time difference. Analyses performed in study subjects defined as normotensive and hypertensive at age 68.

	Normotensive at age 68		Hypertensive at age 68	
	ICA left	ICA right	ICA left	ICA right
	*N* = 55	*N* = 57	*N* = 55	*N* = 59
SBP, age 81	.384^∗∗^	.221	−.007	−.071
DBP, age 81	.102	−.058	−.282^∗^	−.139
SBP, age difference	.303^∗^	.183	.164	−.165
DBP, age difference	.216	.083	−.058	−.033
Pulse pressure, age 81	.373^∗∗^	.274^∗^	−.130	−.019
ABI-left, age 81	−.430^∗∗^	−.072	−.068	−.338^∗^
ABI-right, age 81	−.253^∗^	−.074	−.125	−.448^∗∗^
ABI-left, age 68	−.212	.110	−.075	−.374^∗∗^
ABI-right, age 68	−.254^∗^	.000	−.104	−.171
ABI-left, difference	−.381^∗∗^	−.156	.131	−.127
ABI-right, difference	−.170	−.053	−.034	−.358^∗∗^

^∗^Correlation is significant at  .05 level (2-tailed). ^∗∗^Correlation is significant at  .01 level (2-tailed). ICA: internal carotid artery.

**Table 5:** Distribution of cerebral blood flow values in different brain areas in subjects defined as normo- or hyper-tensive at age 68.

Regional cerebral blood flow	Definition of blood pressure at age 68		
	Normotensives	Hypertensives	*P*
Frontal			
R	81.1 (6.0)	79.9 (5.9)	ns.
L	80.7 (6.1)	79.4 (5.6)	ns.
Mesial temporal			
R	79.5 (4.5)	79.8 (4.9)	ns.
L	78.8 (4.6)	79.4 (4.8)	ns.
Lateral temporal			
R	79.3 (4.5)	78.2 (4.7)	ns.
L	78.7 (5.2)	77.5 (5.4)	ns.
Superior parietal			
R	82.6 (6.9)	80.8 (7.0)	ns.
L	83.8 (8.0)	82.4 (7.0)	ns.
Inferior parietal			
R	81.3 (6.6)	79.4 (5.9)	ns.
L	78.3 (6.8)	77.7 (6.0)	ns.
Basal ganglia			
R	93.3 (6.9)	92.0 (7.0)	ns.
L	91.6 (6.4)	90.4 (7.4)	ns.
Subcortical	64.2 (6.6)	62.5 (6.6)	ns.

ns.: nonsignificant; R: right; L: left.
